# Mind over matter: psychological insights for effective weight reduction through exercise in university students

**DOI:** 10.3389/fpubh.2026.1865508

**Published:** 2026-09-03

**Authors:** Wang Ying, Cong Guo, Meirong Zhang, Yixin Zhao

**Affiliations:** 1 Public Sports Department, Taizhou University, Taizhou, Jiangsu, China; 2 Zhoukou Normal University, Zhoukou, Henan, China; 3 Changzhou Mechanical and Electrical Vocational and Technical College, Changzhou, China

**Keywords:** behavioral change, mental health, mindfulness, motivation, self-determination theory, self-efficacy, weight reduction

## Abstract

This comprehensive review article examines the multifaceted psychological dimensions that underpin effective weight reduction through exercise among university students, a demographic uniquely vulnerable to weight gain and sedentary lifestyles during a critical transitional life period. Adopting a structured narrative (integrative) review approach and drawing on peer-reviewed literature identified through structured searches of PubMed, PsycINFO, Scopus, Web of Science, and ERIC (2000–2025), with emphasis on studies recently published, the present review explores how psychological constructs including self-determination, self-efficacy, goal-setting, mindfulness, and habit formation interact with exercise behavior to facilitate or impede weight management in higher education settings. The article integrates well-established theoretical frameworks such as Self-Determination Theory, Social Cognitive Theory, and the Theory of Planned Behavior, situating them within the specific psychosocial context of university life, including academic stress, peer dynamics, campus environments, and digital technology use. Case studies from various university settings are woven throughout each section to illustrate practical applications of these psychological insights. The review further examines how gender differences, mental health comorbidities, and technology-mediated interventions modulate the effectiveness of exercise-based weight reduction strategies. Findings consistently indicate that psychological readiness, intrinsic motivation, autonomy support, and personalized behavioral strategies are more predictive of sustained weight reduction than exercise prescription alone. The article concludes with evidence-based recommendations for university stakeholders, counselors, and health professionals seeking to design psychologically informed exercise programs that promote lasting weight management and holistic well-being among student populations.

## 1 Introduction

The transition to university life represents one of the most psychologically demanding periods in a young adult’s developmental trajectory, and this period is consistently associated with significant weight gain, declining physical activity levels, and the emergence of unhealthy dietary patterns that can have lasting consequences for health across the lifespan (1, 2). Research indicates that the average university student gains between two and five kilograms during the first year of enrollment alone, a phenomenon popularly known as the “freshman fifteen” that, while often exaggerated in popular media, reflects a genuine epidemiological trend supported by longitudinal data from multiple countries and institutional settings (2, 3). The reasons for this weight gain are multifactorial and include changes in eating habits, increased alcohol consumption, reduced structured physical activity, disrupted sleep patterns, and elevated psychological stress, all of which interact in complex and often synergistic ways to create an obesogenic environment within the university context (4, 5). Yet despite widespread awareness of the health risks associated with excess weight gain, many university students struggle to adopt and sustain regular exercise habits, not because they lack knowledge about the benefits of physical activity, but because they face a constellation of psychological barriers that undermine their motivation, self-regulation, and behavioral consistency (6, 7). Understanding these psychological barriers and the mechanisms through which they operate is essential for designing interventions that go beyond simple exercise prescription to address the cognitive, emotional, and social dimensions of behavior change in this population.

The concept of “mind over matter” in the context of exercise-based weight reduction captures a fundamental truth that has been increasingly validated by behavioral science: that the psychological state of the individual is often a more powerful determinant of exercise adherence and weight management outcomes than the specific type, duration, or intensity of exercise performed (8, 9). This perspective challenges the traditional biomedical model, which has historically emphasized caloric expenditure and physiological adaptations as the primary mechanisms of weight loss, and instead foregrounds the role of motivation, self-perception, emotional regulation, and cognitive appraisal in shaping exercise behavior over time (10, 11). For university students in particular, the psychological dimensions of exercise are especially salient because this population is navigating concurrent developmental tasks including identity formation, social integration, academic achievement, and the establishment of autonomous health behaviors, all of which interact with exercise motivation in dynamic and sometimes contradictory ways (12, 13). A first-year engineering student at a large public university in the southeastern United States, for example, reported in a campus wellness study that she had joined the campus recreation center with the explicit goal of losing weight but abandoned her exercise routine within 3 weeks because the perceived judgment from more experienced exercisers triggered intense social physique anxiety, illustrating how psychological factors can rapidly override initial behavioral intentions even when physical resources are readily available (14, 15).

**Figure 1:**
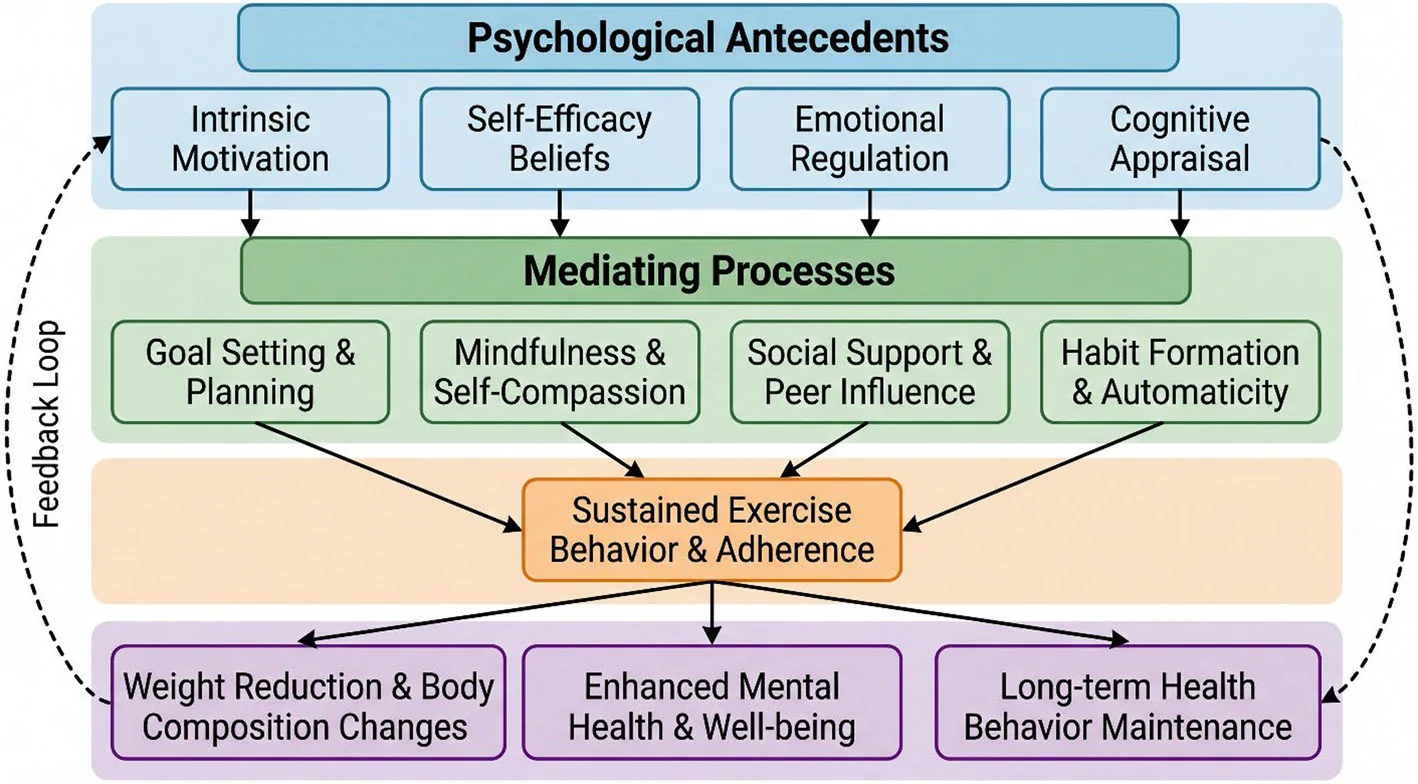
Conceptual model: psychological pathways to exercise-based weight reduction in university students. This figure illustrates the hypothesized relationships between psychological antecedents, mediating processes, sustained exercise behavior, and weight management outcomes, together with barriers to exercise (e.g., academic stress, body-image concerns, low self-efficacy, and mental-health challenges) that attenuate the pathway from psychological antecedents to sustained exercise behavior, and dual feedback loops in which weight-management and psychological outcomes reciprocally influence both the psychological antecedents and the mediating processes.

The present review article aims to provide a comprehensive, evidence-based synthesis of the psychological insights that are most relevant to understanding and promoting effective weight reduction through exercise among university students, drawing on recent literature published from 2020 onwards while also situating these findings within the broader theoretical landscape of health behavior change. The article is organized around several key thematic areas, beginning with an examination of the cognitive and emotional factors that influence exercise behavior in university students, followed by a detailed discussion of the major theoretical frameworks that have been applied to this domain, including Self-Determination Theory, Social Cognitive Theory, and the Theory of Planned Behavior (16–18). Subsequent sections address the specific psychological barriers that university students face in adopting exercise for weight management, the evidence-based psychological strategies that have been shown to enhance exercise adherence and weight loss outcomes, and the role of social, environmental, and technological factors in supporting or undermining these processes (19, 20). Throughout the article, case studies from diverse university settings are integrated within each section and subsection to provide concrete illustrations of how these psychological principles operate in real-world contexts, thereby bridging the gap between theoretical knowledge and practical application. The review also addresses gender differences in psychological responses to exercise-based weight management and examines the long-term psychological outcomes associated with sustained exercise behavior, before concluding with recommendations for researchers, practitioners, and university administrators seeking to create supportive environments for student health and well-being. Figure 1 presents the conceptual model that organizes this review, mapping psychological antecedents, mediating processes, barriers to exercise, sustained exercise behavior, and weight-management outcomes, together with feedback loops that link outcomes back to both the antecedents and the mediating processes.

### 1.1 Scope and review approach

This article is a structured narrative (integrative) review rather than a systematic review or meta-analysis, and it is reported with reference to recognized guidance for narrative and integrative syntheses (21–23). Relevant literature was identified through structured searches of five electronic databases (PubMed, PsycINFO, Scopus, Web of Science, and ERIC) covering the period 2000–2025, with emphasis on peer-reviewed work published from 2020 onward. Search terms combined population descriptors (e.g., “university students,” “college students,” “young adults,” “higher education”) with psychological and behavioral descriptors (e.g., “exercise,” “physical activity,” “weight management,” “self-determination,” “self-efficacy,” “motivation,” “goal setting,” “mindfulness,” “habit formation,” “body image”) using Boolean operators, and the reference lists of key articles were hand-searched to identify additional sources.

The search returned a combined total of approximately 920 records across databases; after removal of duplicates (*n* = 340) and screening of titles and abstracts for relevance to the review scope, 185 full-text articles were assessed, and 115 met inclusion criteria and are cited in this review. Sources were included if they were peer-reviewed, published in English, and addressed psychological determinants of exercise or weight management in university-aged or young-adult populations; foundational conceptual and theoretical works underpinning the frameworks discussed were also retained. Editorials, non-peer-reviewed material, and studies unrelated to psychological processes were excluded. Because this is a narrative rather than a quantitative synthesis, the strength and consistency of the evidence were appraised qualitatively [informed by SANRA; (21)] rather than through formal meta-analytic pooling; accordingly, no pooled or statistically weighted effect sizes are reported. To preserve anonymity and to illustrate mechanisms concisely, the case examples presented throughout this review are best understood as illustrative composite vignettes that synthesize patterns commonly reported in the cited literature; any numerical values within them are approximate and are provided for illustration rather than as primary empirical findings. Consistent with the aims of a narrative review, the synthesis gives explicit attention to inconsistent, limited, or mixed evidence as well as to supportive findings, and it does not claim exhaustive coverage of the literature.

## 2 The psychology of weight management in university students

The psychology of weight management among university students is a richly layered phenomenon that cannot be adequately understood through any single disciplinary lens, as it encompasses cognitive processes such as attention, memory, and decision-making alongside emotional experiences including anxiety, shame, pride, and joy, all of which interact with social and environmental factors to produce highly individualized patterns of exercise behavior and weight change (24, 25). University students represent a particularly interesting population for studying the psychology of weight management because they are at a stage of life where health behaviors are being established that will often persist into middle and later adulthood, and because the university environment provides a concentrated microcosm of the social, cultural, and institutional influences that shape health behavior more broadly (26, 27). Research from a longitudinal study conducted across three European universities found that students who entered university with positive exercise identities and strong self-regulatory skills were significantly more likely to maintain healthy body weight throughout their 4 years of study, compared to peers who entered with similar baseline fitness levels but weaker psychological resources, suggesting that psychological factors may be more predictive of long-term weight outcomes than initial physical conditioning (28, 29). Understanding these psychological factors requires an examination of both the cognitive structures that guide exercise-related thinking and the emotional and affective processes that color the subjective experience of physical activity, as these two domains are deeply interconnected and mutually reinforcing in ways that have significant implications for intervention design and delivery.

### 2.1 Cognitive factors influencing exercise behavior

Cognitive factors play a central and often underappreciated role in determining whether university students adopt, maintain, or abandon exercise behaviors aimed at weight reduction, and these factors operate at multiple levels ranging from basic attentional processes and information processing to higher-order constructs such as beliefs, attitudes, expectations, and self-regulatory strategies (30, 31). At the most fundamental level, the way students think about exercise, including their beliefs about its effectiveness for weight loss, their expectations about how quickly results will appear, and their interpretations of their own exercise experiences, powerfully shapes their motivation to continue exercising even in the face of competing demands and setbacks (15, 32). Research consistently demonstrates that university students who hold unrealistic expectations about the speed and magnitude of weight loss from exercise are significantly more likely to abandon their exercise programs prematurely, because the inevitable discrepancy between expected and actual outcomes triggers cognitive dissonance, frustration, and a sense of futility that undermines ongoing motivation (10, 33). For instance, a case study documented at a mid-sized Canadian university involved a second-year business student who began a rigorous five-day-per-week exercise program with the expectation of losing 10 kg within the first month, and when this unrealistic goal was not met, he concluded that exercise was ineffective for his body type and ceased all physical activity, despite having actually improved his cardiovascular fitness and reduced his body fat percentage by a measurable amount (34). This case illustrates the critical importance of cognitive restructuring in exercise interventions, as it demonstrates how maladaptive thought patterns can lead to behavioral disengagement even when objective progress is being made.

Self-efficacy, defined by Bandura (17, 35) as the belief in one’s capacity to execute the behaviors required to produce specific outcomes, is arguably the single most influential cognitive factor in exercise-based weight management among university students, as it affects not only whether individuals initiate exercise but also how much effort they expend, how long they persist in the face of difficulties, and how they interpret and respond to setbacks and failures. Multiple studies have demonstrated that exercise self-efficacy is a strong and consistent predictor of physical activity levels among university students across diverse cultural and institutional contexts, and that interventions designed to enhance self-efficacy through mastery experiences, social modeling, verbal persuasion, and the reinterpretation of physiological arousal can significantly increase both the frequency and intensity of exercise participation in this population (15, 36, 37). The role of self-efficacy is particularly important during the early stages of an exercise program, when students are most vulnerable to dropout, because initial experiences of success or failure during this period can create self-reinforcing cycles of either increasing confidence and engagement or decreasing confidence and withdrawal (17, 38). A research group at an Australian university documented the case of a group of sedentary first-year students who participated in a structured six-week exercise introduction program that was specifically designed to build self-efficacy through graduated task difficulty, supportive feedback, and peer modeling; the results showed that students in this program not only exercised more frequently during the intervention period but also maintained significantly higher activity levels at a 6-month follow-up, compared to students who received standard exercise advice without the self-efficacy enhancement component (39, 40). Outcome expectations, which refer to the anticipated consequences of performing a behavior, also play a critical role in shaping exercise motivation, as students who expect exercise to produce not only weight loss but also psychological benefits such as improved mood, reduced stress, and enhanced social connection are more likely to sustain their exercise behavior over time than students whose expectations are focused exclusively on physical appearance changes (36, 41). Cognitive flexibility, or the ability to adapt one’s thinking in response to changing circumstances, is another important cognitive factor that has received increasing attention in the exercise psychology literature, as university students who demonstrate greater cognitive flexibility are better able to adjust their exercise plans when faced with schedule disruptions, injury, or other obstacles, rather than adopting an all-or-nothing approach that leads to complete behavioral disengagement when perfect adherence is not possible (42, 43).

### 2.2 Emotional and affective determinants of exercise behavior

The emotional and affective dimensions of exercise are increasingly recognized as critical determinants of exercise behavior among university students, and this recognition has led to a growing body of research examining how feelings experienced before, during, and after exercise influence subsequent motivation, adherence, and weight management outcomes in this population (44, 45). Affect, which encompasses both discrete emotions such as happiness, anxiety, guilt, and pride and broader mood states such as feeling energized or fatigued, operates as a powerful motivational signal that can either attract students toward or repel them away from exercise, often in ways that override conscious intentions and rational decision-making processes (46, 47). The affective response to exercise is highly variable across individuals and is influenced by factors including exercise intensity, duration, context, social environment, and the individual’s psychological history, which means that a one-size-fits-all approach to exercise prescription is likely to be less effective than an approach that takes into account each student’s unique affective profile and tailors exercise recommendations accordingly (48, 109). Research conducted at a large Scandinavian university followed a cohort of 340 undergraduate students over one academic year and found that the single strongest predictor of exercise maintenance at the end of the year was not exercise intensity, duration, or type, but rather the degree of positive affect that students reported experiencing during their exercise sessions, suggesting that enjoyment may be a more important target for intervention than traditional physiological variables such as heart rate zones or caloric expenditure (49, 50).

Emotional regulation, defined as the processes by which individuals influence which emotions they have, when they have them, and how they experience and express them, is particularly relevant to exercise-based weight management in university students because this population is exposed to high levels of academic, social, and financial stress that can trigger emotional eating, sedentary coping behaviors, and avoidance of physical activity (25, 51). Students who lack effective emotional regulation skills may turn to food rather than exercise as a means of managing negative emotions, creating a vicious cycle in which stress leads to overeating, overeating leads to weight gain, weight gain leads to reduced self-esteem and increased body dissatisfaction, and these negative self-perceptions further reduce the likelihood of engaging in physical activity (52, 53). Conversely, students who learn to use exercise as a form of emotional regulation, recognizing that physical activity can serve as a healthy outlet for stress, frustration, and anxiety, tend to develop more sustainable exercise habits because they experience exercise as intrinsically rewarding rather than merely instrumentally useful for weight control (54, 55). A particularly instructive case was documented at a Korean university where a group of female students who had been identified as emotional eaters participated in a 10-week program that combined moderate-intensity aerobic exercise with psychoeducational workshops on emotional awareness and regulation; by the end of the program, participants reported significant reductions in emotional eating episodes, improved mood regulation, and modest but meaningful weight loss, and crucially, these benefits were maintained at a three-month follow-up, suggesting that the integration of psychological and physical components produced more durable outcomes than either component alone would have achieved (40, 55). The concept of exercise-induced affect regulation has also been linked to broader constructs such as psychological resilience and well-being, with several studies indicating that university students who exercise regularly report not only better mood and lower stress but also greater overall life satisfaction and a stronger sense of personal agency, all of which serve as protective factors against the psychological challenges that commonly accompany weight management efforts during the university years (56, 57).

## 3 Theoretical frameworks for understanding exercise motivation

Understanding why university students choose to exercise or not exercise, and why some students sustain their exercise behavior over time while others relapse into sedentary lifestyles, requires the application of theoretical frameworks that can account for the complex interplay between cognitive, affective, social, and environmental factors that shape behavior in this population (9, 58). Several major psychological theories have been applied to the study of exercise motivation and behavior change in university settings, and while no single theory provides a complete account of the phenomena involved, each offers unique insights that can inform the design of more effective interventions for exercise-based weight management (30, 59). Table 1 presents a comparative overview of the three most influential theoretical frameworks discussed in this section. The following subsections examine the three most influential theoretical frameworks in this domain, Self-Determination Theory, Social Cognitive Theory, and the Theory of Planned Behavior, with a focus on how each theory has been applied to understand and promote exercise for weight reduction among university students specifically, and with illustrative case studies that demonstrate the practical relevance of each framework in real-world university contexts.

**Table 1:** Comparison of major theoretical frameworks applied to exercise motivation in university students.

Framework	Core constructs	Key predictions for exercise	Strengths and limitations
Self-determination theory (60)	Autonomy, competence, relatedness; intrinsic vs. extrinsic motivation continuum	Intrinsic motivation leads to greater exercise persistence; need satisfaction enhances autonomous motivation and well-being	Strong empirical support; explains quality of motivation. Limited guidance on how to address structural barriers
Social cognitive theory (35)	Self-efficacy, outcome expectations, self-regulation, observational learning, reciprocal determinism	Higher self-efficacy predicts exercise initiation and maintenance; modeling and mastery experiences build confidence	Highly practical and actionable. May underestimate emotional and unconscious factors in behavior
Theory of planned behavior (16)	Attitudes, subjective norms, perceived behavioral control, behavioral intention	Intentions predict exercise behavior; perceived behavioral control moderates the intention-behavior relationship	Parsimonious and widely tested. Intention-behavior gap limits predictive utility for actual exercise

### 3.1 Self-determination theory and exercise adherence

Self-Determination Theory (SDT), originally developed by Deci and Ryan (60), has emerged as one of the most widely applied and empirically supported theoretical frameworks for understanding exercise motivation in university student populations, and its emphasis on the quality rather than merely the quantity of motivation makes it particularly well-suited to explaining why some students maintain long-term exercise habits while others abandon their efforts despite initial enthusiasm (9, 61). At the core of SDT is the distinction between intrinsic motivation, which refers to engaging in an activity because it is inherently enjoyable, interesting, or satisfying, and extrinsic motivation, which refers to engaging in an activity because of external pressures, rewards, or contingencies, with the theory positing that intrinsic motivation is associated with greater behavioral persistence, psychological well-being, and performance quality than extrinsic motivation across virtually all domains of human activity (18, 62). In the context of exercise-based weight management, this distinction has profound implications, because it suggests that students who exercise primarily because they enjoy the experience of physical activity and find it personally meaningful are more likely to sustain their exercise behavior and achieve lasting weight reduction than students who exercise primarily because they feel pressured to lose weight by external sources such as social media, peers, family members, or health professionals (63, 64).

choices regarding exercise type and intensity (autonomy support), provide non-comparative positive feedback (competence support), and foster a welcoming and inclusive social atmosphere (relatedness support); the redesigned program showed a substantial increase in student retention over the previous year’s program, and participants reported significantly higher levels of intrinsic motivation and exercise enjoyment, demonstrating the practical value of theory-informed program design (59, 65). The concept of internalization, which in SDT refers to the process by which externally motivated behaviors become progressively more self-endorsed and integrated into one’s sense of identity, is particularly relevant to understanding how university students can transition from exercising for weight loss (an extrinsic motive) to exercising because physical activity has become an integral and valued part of who they are as a person, and research suggests that this internalization process is facilitated by the consistent satisfaction of autonomy, competence, and relatedness needs over time (9, 66). A meta-analysis of SDT-informed exercise interventions published by Ntoumanis et al. (59) found that interventions incorporating all three need-supportive components produced significantly larger effects on physical activity behavior, autonomous motivation, and psychological health than interventions that targeted only one or two needs, underscoring the importance of addressing the full spectrum of basic psychological needs in exercise programs for university students.

Framed more broadly, the practical implication of Self-Determination Theory is not simply to provide autonomy support but to deliver need-supportive instruction that simultaneously supports students’ autonomy, competence, and relatedness. In applied university settings, autonomy support includes offering meaningful choices over exercise mode, intensity, and timing, providing clear rationales for recommended activities, and using non-controlling, invitational language; competence support includes structuring optimal challenges, scaffolding progression, and giving specific informational (rather than normative or comparative) feedback; and relatedness support includes cultivating inclusive, non-judgmental exercise climates and deliberately fostering peer connection. Longitudinal evidence indicates that the satisfaction of all three needs predicts more adaptive motivational development over time rather than only concurrent engagement; for example, Yun et al. (67) used latent growth modeling to show that psychological-need satisfaction predicted a positive developmental trajectory of motivation in young people, reinforcing the value of comprehensive need-supportive instruction over narrower autonomy-only approaches (59, 68). At the same time, the evidence is not uniform: effects vary by context and measurement, and the relative contribution of each need to sustained exercise remains an open question, underscoring the need for cautious interpretation.

### 3.2 Social cognitive theory and self-efficacy in exercise contexts

**Figure 2:**
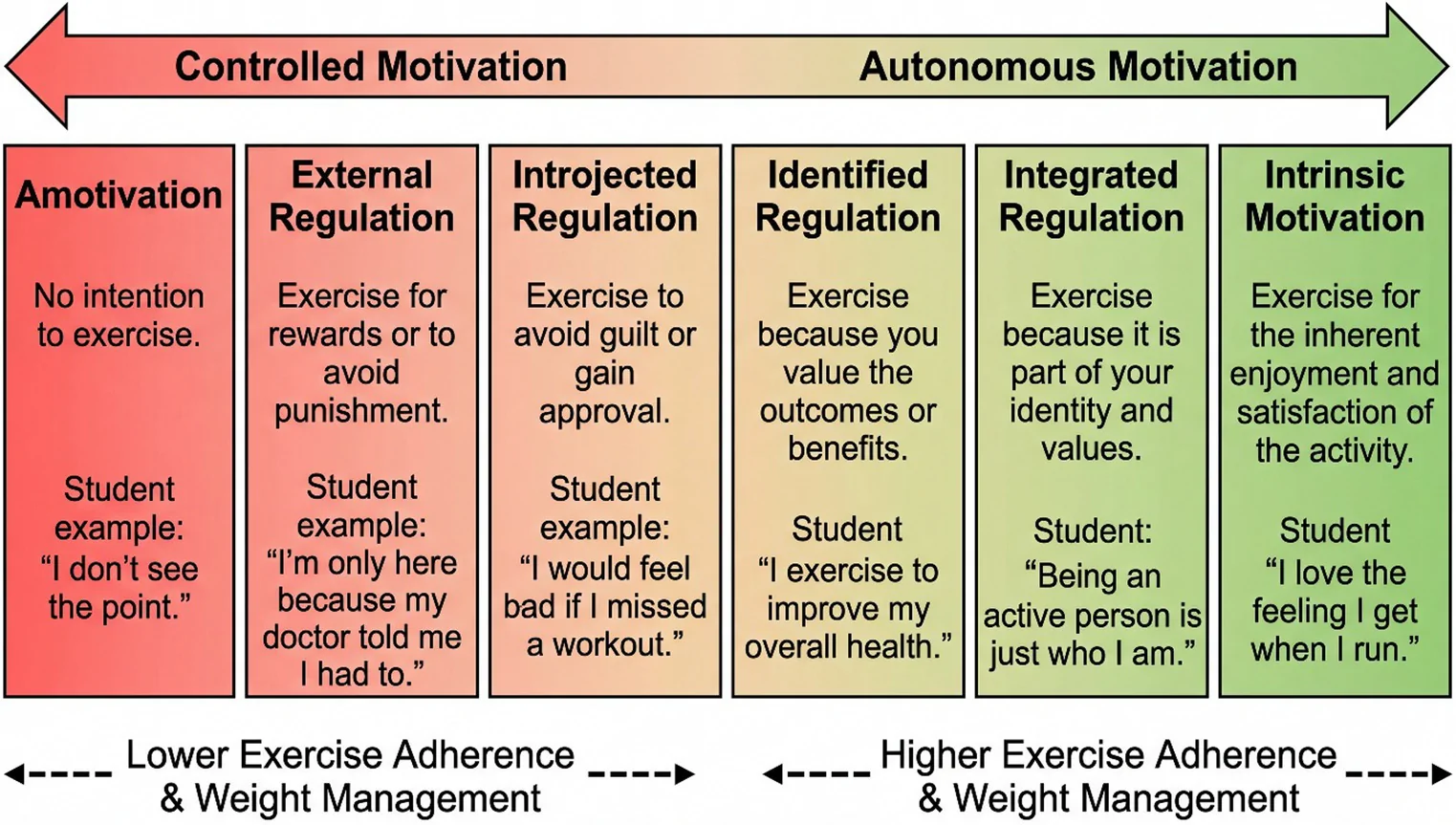
Self-determination theory: motivation continuum for exercise behavior. This figure depicts the continuum from amotivation through external, introjected, identified, and integrated regulation to intrinsic motivation, illustrating how different types of motivation relate to exercise adherence and weight management outcomes in university students.

Social cognitive theory (SCT), developed by Bandura (17, 35), provides a complementary perspective on exercise motivation by emphasizing the dynamic and reciprocal interactions between personal cognitive factors, behavioral patterns, and environmental influences, a concept known as triadic reciprocal determinism that captures the reality that exercise behavior is simultaneously shaped by and shapes the contexts in which it occurs (17, 31). Within SCT, self-efficacy is identified as the central mechanism of human agency, and as discussed in the previous section on cognitive factors, exercise self-efficacy is consistently one of the strongest predictors of physical activity behavior among university students, with higher self-efficacy associated with greater exercise initiation, more persistent effort, more effective recovery from setbacks, and better weight management outcomes (15, 37). However, SCT goes beyond self-efficacy to also emphasize the role of observational learning, or modeling, as a means through which individuals acquire new behaviors, with research indicating that university students who observe peers successfully engaging in exercise are more likely to develop positive exercise attitudes, higher self-efficacy expectations, and greater behavioral intentions than students who lack exposure to such models (17, 69) (see Figure 2).

The concept of self-regulation, which in SCT refers to the process by which individuals monitor their behavior, compare it to personal standards, and adjust their actions accordingly, is particularly important for exercise-based weight management because sustained weight reduction requires ongoing behavioral monitoring and adjustment that extends far beyond the initial decision to begin exercising (31, 32). University students often struggle with self-regulation because the academic environment places heavy demands on their cognitive resources, leaving less capacity for the planning, monitoring, and evaluation processes that effective self-regulation requires, and because the relatively unstructured nature of university life provides fewer external supports for behavioral consistency than the more structured environments they may have experienced during high school (43, 70). A case study from a private university in the northeastern United States demonstrated the practical implications of SCT-informed self-regulation training for weight management: a group of 20 overweight sophomore and junior students participated in a semester-long program that taught them to set specific exercise goals, self-monitor their daily activity using wearable devices, identify and address barriers to adherence, and use self-reward strategies to reinforce positive behaviors; at the end of the semester, participants had not only increased their weekly exercise duration by an average of 120 min but had also developed more robust self-regulatory habits that were associated with continued exercise at a 12-month follow-up, even after the formal program had ended (39, 71). Outcome expectations, another key construct in SCT, refer to the anticipated positive and negative consequences of engaging in a behavior, and research with university students indicates that those who hold positive outcome expectations for exercise across multiple domains, including physical health, mental well-being, social connection, and academic performance, are significantly more likely to exercise regularly than those whose outcome expectations are limited to a single domain such as weight loss or physical appearance (36, 72). SCT’s emphasis on the environmental determinants of behavior, including the availability of exercise facilities, the presence of exercise role models, and the social norms surrounding physical activity within a given university culture, also highlights the importance of structural and institutional changes in supporting individual behavior change, a point that is often overlooked in interventions that focus exclusively on individual-level psychological factors (73, 74).

### 3.3 Theory of planned behavior and the intention-behavior gap

The Theory of Planned Behavior (TPB), originally proposed by Ajzen (16) and extensively refined in subsequent decades, provides a parsimonious yet powerful framework for understanding exercise intentions and behavior among university students by positing that behavioral intention, the most proximal predictor of behavior, is determined by three factors: attitudes toward the behavior, subjective norms (perceived social pressure to perform or not perform the behavior), and perceived behavioral control (the individual’s perception of their ability to perform the behavior given available resources and anticipated obstacles) (16, 30). In the context of exercise-based weight reduction, the TPB suggests that university students are most likely to form strong intentions to exercise when they hold positive attitudes toward physical activity, perceive that important others (peers, family members, instructors) support their exercise behavior, and believe that they have the necessary skills, time, and resources to exercise regularly, and a substantial body of empirical research has confirmed these predictions across diverse student populations (58, 75).

However, one of the most important and well-documented findings in TPB research is the existence of an “intention-behavior gap,” the phenomenon whereby many individuals who form strong intentions to exercise nonetheless fail to translate those intentions into consistent behavior, and this gap is particularly pronounced among university students, who often report high levels of exercise intention but substantially lower levels of actual exercise behavior (7, 76). Research suggests that the intention-behavior gap in exercise is influenced by a range of moderating factors including self-regulatory capacity, habit strength, action planning, environmental cues, and competing behavioral demands, and that closing this gap requires interventions that go beyond merely strengthening intentions to also provide students with the specific self-regulatory tools and environmental supports needed to convert good intentions into consistent action (76, 77). A particularly well-documented case study at a large German university examined the implementation of an intervention designed to bridge the intention-behavior gap among students who had expressed strong exercise intentions but were not currently exercising regularly; the intervention required participants to form specific implementation intentions (detailed “if-then” plans specifying when, where, and how they would exercise), and results showed that participants who formed implementation intentions were significantly more likely to exercise at their intended frequency over the following 8 weeks compared to a control group that formed only general goal intentions, with the implementation intention group also showing greater weight loss at the end of the intervention period (38, 78). This finding has been replicated in multiple university settings and supports the broader theoretical proposition that effective exercise-based weight management requires not only motivation (wanting to exercise) but also volition (the capacity to translate motivation into planned and self-regulated action), a distinction that has been increasingly emphasized in contemporary models of health behavior change (75, 76). The TPB has also been used to identify subgroups of university students who may require different intervention approaches based on their specific profiles of attitudes, norms, and perceived behavioral control, with research suggesting that students whose exercise barriers are primarily attitudinal (negative beliefs about exercise) may benefit most from cognitive restructuring and motivational enhancement strategies, while students whose barriers are primarily normative (lack of social support) or control-related (perceived lack of time or resources) may benefit more from social networking interventions or environmental modifications, respectively (30, 58).

## 4 Psychological barriers to exercise among university students

**Figure 3:**
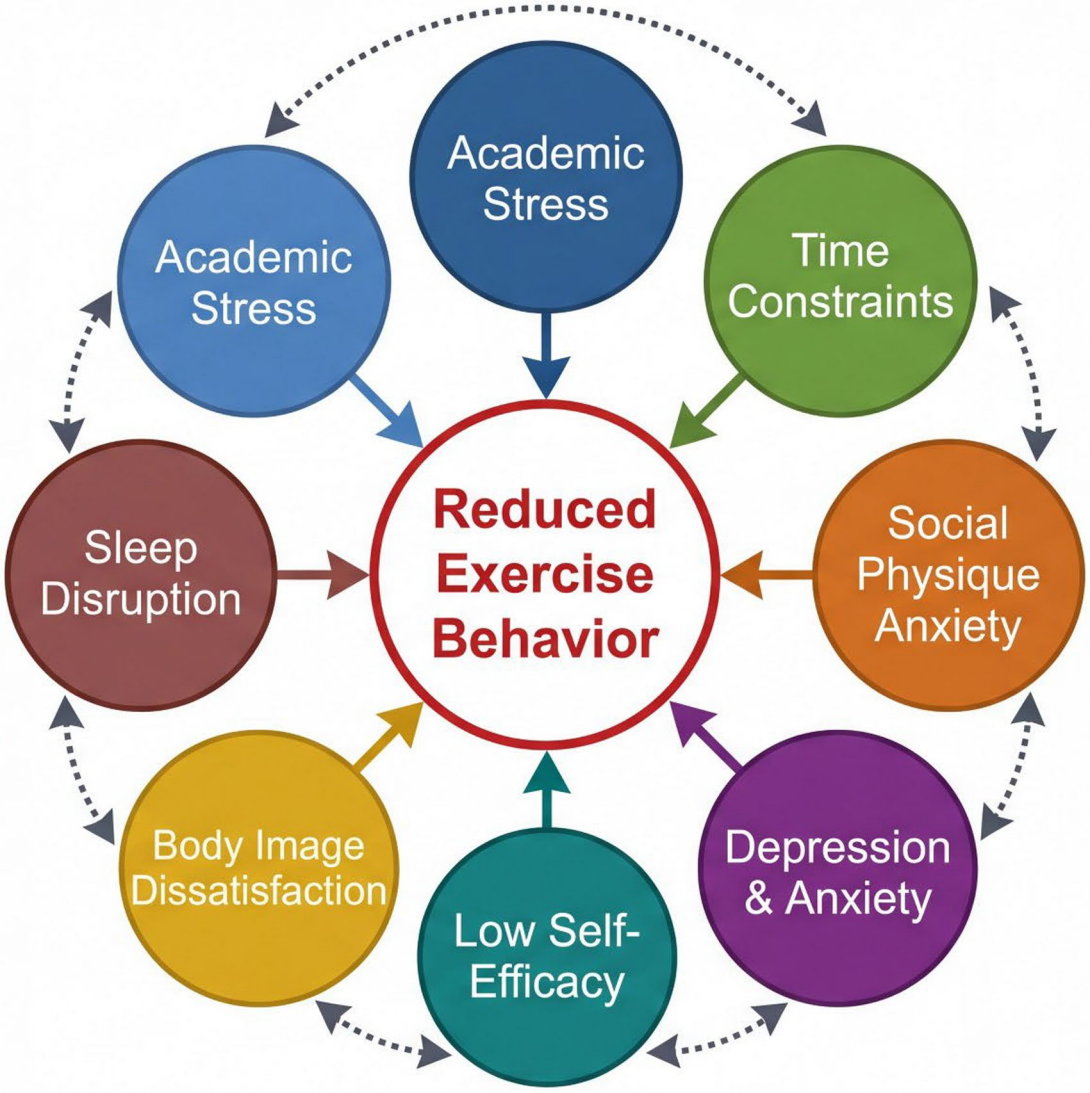
Interconnected psychological barriers to exercise in university students. This diagram illustrates how multiple psychological barriers simultaneously influence exercise avoidance, with dotted lines indicating bidirectional reinforcement between barrier categories.

Despite the well-documented physical and psychological benefits of regular exercise, a substantial proportion of university students fail to meet minimum physical activity guidelines, and this behavioral deficit is driven not primarily by a lack of awareness or knowledge about the importance of exercise but rather by a complex set of psychological barriers that vary in salience and impact across individual students, institutional contexts, and cultural settings (29, 79). Understanding these psychological barriers is essential for developing targeted and effective interventions, because strategies that address the wrong barriers for a given population or individual are unlikely to produce meaningful behavioral change, and may even be counterproductive if they inadvertently increase resistance or undermine motivation (19, 80). The following subsections examine three categories of psychological barriers that have been most consistently identified in the literature as impediments to exercise-based weight management among university students: academic stress and time constraints, body image concerns and exercise avoidance, and mental health challenges. Table 2 summarizes these barriers, their psychological mechanisms, and the subgroups they most affect, and Figure 3 depicts how the barriers interact and mutually reinforce one another.

**Table 2:** Summary of psychological barriers to exercise-based weight management among university students.

Barrier category	Psychological mechanisms	Impact on exercise	Affected subgroups
Academic stress	Ego depletion; perceived time scarcity; self-regulatory resource drain; cognitive overload	Substantial decrease in physical activity during exam periods (reported as roughly two-thirds in illustrative vignettes); exercise viewed as time-luxury	STEM students; medical/law students; students with high academic anxiety
Body image concerns	Social physique anxiety; social comparison; internalized weight stigma; appearance-based self-evaluation	Avoidance of gym/group settings; exercise dropout; development of maladaptive exercise patterns	Female students; higher-BMI students; exercise beginners; heavy social media users
Depression and anxiety	Low energy and motivation; anhedonia; social withdrawal; misinterpretation of arousal as exercise intolerance	Complete exercise cessation; reduced initiation capacity; avoidance of social exercise settings	Students with pre-existing mental health conditions; students in high-stress transition periods
Low self-efficacy	Negative past exercise experiences; lack of role models; insufficient mastery experiences; negative self-talk	Failure to initiate exercise programs; premature dropout; low effort during exercise	First-generation students; students new to structured exercise; students with chronic health conditions
Sleep disruption	Circadian rhythm disruption; fatigue-related cognitive impairment; reduced decision-making capacity	Decreased exercise frequency and intensity; substitution of exercise time with sleep	Night-shift workers; students with irregular schedules; students with insomnia

### 4.1 Academic stress and time constraints

Academic stress and the perception of insufficient time are among the most frequently cited barriers to exercise among university students, and these two factors are closely interrelated because the experience of academic pressure often distorts students’ perception of available time, leading them to overestimate the amount of time they spend studying and underestimate the amount of time that could realistically be allocated to physical activity without compromising academic performance (49, 70). Research from a multi-site study involving over 2,500 university students across eight institutions found that perceived academic stress was significantly negatively correlated with physical activity levels, and that this relationship was partially mediated by perceived time constraints, suggesting that stress influences exercise behavior at least in part by creating a subjective sense of temporal scarcity that makes exercise seem like an unaffordable luxury rather than a necessary investment in health and productivity (24, 51). Paradoxically, the students who feel most stressed and most pressed for time are often the ones who would benefit most from regular exercise, as a robust body of evidence demonstrates that physical activity reduces perceived stress, improves cognitive function, enhances sleep quality, and increases academic performance, creating a positive feedback loop that can actually free up cognitive resources and subjective time for studying (49, 81).

The relationship between academic stress and exercise avoidance is further complicated by the phenomenon of ego depletion, a concept from self-regulation theory suggesting that effortful cognitive activities (such as studying for exams or writing papers) consume a limited pool of self-regulatory resources, leaving less capacity for other demanding behaviors, including exercise, which also requires self-regulation for initiation and maintenance (32, 43). A detailed case study from a Japanese university followed a group of medical students through their examination periods and found that physical activity levels dropped substantially by roughly two thirds in this illustrative vignette during the 2 weeks preceding major exams compared to non-exam periods, and that this decline was most pronounced among students who reported the highest levels of academic anxiety, suggesting that the psychological burden of exam stress created a double barrier by simultaneously increasing the perceived cost of exercising (time away from studying) and reducing the self-regulatory capacity needed to initiate exercise (12, 51). However, the same study also identified a subgroup of students who maintained or even increased their exercise levels during exam periods, and qualitative interviews with these students revealed that they had developed robust exercise habits that required minimal conscious self-regulation, highlighting the importance of habit formation as a psychological strategy for protecting exercise behavior against the erosive effects of academic stress (42, 82). These findings suggest that interventions aimed at helping university students exercise for weight management should prioritize the early establishment of exercise habits during less stressful periods of the academic calendar, so that these habits can sustain exercise behavior during the more demanding periods when self-regulatory resources are depleted and the temptation to abandon exercise is greatest.

### 4.2 Body image concerns and exercise avoidance

Body image concerns represent a paradoxical and psychologically complex barrier to exercise-based weight management among university students, because the very dissatisfaction with one’s body that often motivates the initial desire to lose weight can simultaneously create anxiety about exercising in social settings, leading to avoidance of the physical activity that is necessary to achieve the desired body composition changes (14, 83). Social physique anxiety, defined as the anxiety experienced in response to the perception that others are evaluating one’s physique, is prevalent among university students of all body types and is particularly inhibiting in exercise contexts where bodies are on display, such as fitness centers, group exercise classes, swimming pools, and outdoor running routes, where the potential for unfavorable social comparison is heightened (14, 53). Research conducted at multiple universities has consistently found that social physique anxiety is negatively associated with exercise frequency and that this relationship is particularly strong among female students, students with higher body mass indices, and students who are new to exercise, suggesting that the populations who are most in need of exercise for weight management are also the most psychologically vulnerable to the social evaluative aspects of exercise environments (41, 83).

The role of body image in exercise behavior is further complicated by the influence of social media, which has become a pervasive and often toxic force in shaping body ideals and self-perceptions among university-age individuals, with research indicating that frequent exposure to idealized body images on platforms such as Instagram, TikTok, and fitness influencer content is associated with increased body dissatisfaction, heightened social comparison, and more negative attitudes toward one’s own exercise abilities and body composition (52, 53). A case study from a large public university in the Midwestern United States documented the experience of a male sophomore who had been consistently exercising and managing his weight successfully until he began following several fitness influencers on social media; exposure to these highly curated and often unrealistic body standards led to a progressive increase in body dissatisfaction and exercise-related anxiety, eventually resulting in the development of exercise addiction characterized by compulsive overtraining, restrictive eating, and significant psychological distress, illustrating how body image concerns can not only prevent exercise initiation but can also distort exercise behavior in harmful directions even among students who are already physically active (34, 53). Weight stigma, which refers to the social devaluation and discrimination experienced by individuals perceived to be overweight, is another important dimension of the body image barrier, as research consistently shows that experiences of weight stigma are associated with reduced exercise motivation, increased avoidance of physical activity settings, greater comfort eating, and poorer weight management outcomes, creating a cycle of stigma and weight gain that is extremely difficult to break without targeted psychological intervention (33, 53). Interventions that address body image concerns as part of a comprehensive exercise-based weight management program have shown promising results, with approaches including self-compassion training, body functionality appreciation exercises, cognitive defusion techniques, and the creation of “body-safe” exercise spaces that minimize social evaluation and emphasize enjoyment and competence rather than appearance being associated with reduced exercise avoidance and improved weight management outcomes among university students (13, 83).

### 4.3 Mental health challenges as barriers to exercise

Mental health challenges including depression, anxiety, and chronic stress constitute significant psychological barriers to exercise-based weight management among university students, and the prevalence of these conditions in student populations has been increasing steadily in recent years, with surveys consistently indicating that a majority of university students report experiencing clinically significant levels of at least one mental health symptom during their time in higher education (25, 79). Depression is particularly detrimental to exercise behavior because its core symptoms, including low energy, reduced motivation, diminished pleasure in activities, social withdrawal, and feelings of hopelessness, directly counteract the psychological resources that are needed to initiate and maintain regular physical activity, creating a situation where the students who could potentially benefit most from the antidepressant effects of exercise are the least psychologically equipped to access those benefits (54, 55). Anxiety, including both generalized anxiety and specific forms such as social anxiety and performance anxiety, can similarly impair exercise behavior by generating avoidance of novel or socially evaluative situations, increasing physiological arousal that may be misinterpreted as exercise intolerance, and consuming cognitive resources that are needed for exercise planning and self-regulation (57, 84).

Research from a large-scale survey of university students across 24 countries found that students with higher levels of depressive symptoms reported significantly lower levels of physical activity and higher levels of sedentary behavior, and that this relationship remained significant after controlling for demographic variables, academic demands, and physical health status, suggesting that the mental health-exercise relationship is robust and not simply an artifact of other confounding factors (50, 55). A case study from a university mental health service in Ireland described the experience of a final-year psychology student who had been referred for depression and who reported having completely abandoned her previous exercise routine over the preceding 8 months; the clinical team implemented a graded exercise program that began with short daily walks and very gradually increased in duration and intensity, combined with behavioral activation techniques that helped the student identify and challenge the depressive cognitions that were preventing her from exercising; over a 12-week period, the student progressed from five-minute walks to 30-min jogs, reported significant improvements in mood and energy, and achieved a modest weight reduction that she attributed as much to the psychological benefits of re-engaging with physical activity as to the caloric expenditure involved (48, 54). This case illustrates a critical principle in working with mentally distressed university students: that exercise interventions must be calibrated to the individual’s current psychological capacity and must address mental health barriers directly rather than assuming that providing exercise opportunities will be sufficient to overcome the motivational and cognitive impairments associated with psychiatric symptoms. The concept of dual-benefit interventions, which simultaneously target mental health symptoms and exercise behavior through integrated psychological and physical programming, has gained increasing traction in university health services, with evidence suggesting that such integrated approaches produce better outcomes for both mental health and weight management than either psychological treatment or exercise prescription delivered in isolation (48, 55, 85).

## 5 Evidence-based psychological strategies for exercise-induced weight reduction

Having examined the psychological barriers that impede exercise-based weight management among university students, this section turns to the evidence-based psychological strategies that have been shown to enhance exercise adherence and weight reduction outcomes in this population, with a focus on approaches that are grounded in behavioral science, supported by empirical evidence, and feasible for implementation within university settings (19, 64). These strategies are not mutually exclusive, and indeed the most effective interventions typically combine multiple psychological techniques within an integrated framework that addresses the cognitive, emotional, social, and behavioral dimensions of exercise simultaneously, recognizing that the complexity of human behavior requires multifaceted solutions rather than simple prescriptions (8, 59) (see Table 3).

**Table 3:** Evidence-based psychological strategies: mechanisms and implementation approaches.

Strategy	Primary mechanisms	Implementation in university settings	Evidence of effectiveness
Goal setting and implementation intentions	Bridges intention-behavior gap; creates mental links between cues and actions; enhances self-regulation	Structured workshops; counselor-guided SMART goals; if-then planning for specific exercise sessions	Significant increases in exercise frequency and favorable body composition changes in RCTs (38)
Motivational interviewing	Resolves ambivalence; builds autonomous motivation; satisfies basic psychological needs through empathic relational context	Individual counseling; group workshops; peer-led interventions; integrated into campus health services	Greater PA increases, higher autonomous motivation, greater BMI reduction vs. health education (8)
Mindfulness and self-compassion	Enhances body awareness; reduces emotional reactivity; promotes non-judgmental acceptance; buffers against self-criticism	Mindful walking programs; meditation apps; self-compassion workshops; integrated mind–body classes	Improved mood, body satisfaction, and exercise maintenance at 4-week follow-up (91)
Behavioral activation and habit formation	Action before motivation; environmental cue linking; gradual automatization; reduced dependence on conscious self-regulation	Structured scheduling; cue identification; consistent context repetition; non-food rewards; habit-tracking tools	10/12 participants achieved exercise automaticity; significant fitness and body composition improvements at 6 months (82)
Need-supportive instruction	Satisfies autonomy, competence, and relatedness needs; promotes internalization of exercise motivation	Meaningful choices and rationales; non-controlling language; structured optimal challenges; informational (non-comparative) feedback; inclusive, connection-fostering climate; instructor training in autonomy, competence, and relatedness support	Substantial increase in program retention; significant increases in intrinsic motivation (59)

### 5.1 Goal setting and implementation intentions

Goal setting and implementation intentions are among the most extensively researched and consistently effective psychological strategies for promoting exercise behavior and weight management among university students, and their effectiveness derives from their ability to bridge the well-documented gap between behavioral intentions and actual behavior by translating abstract motivational states into concrete, actionable plans that specify precisely what, when, where, and how exercise will be performed (76, 78, 86). The goal-setting literature, synthesized in Locke and Latham's (86) influential goal-setting theory, demonstrates that goals that are specific, challenging but attainable, and accompanied by feedback about progress produce significantly higher levels of performance and behavioral persistence than vague or easy goals, and this principle has been consistently confirmed in exercise and weight management research with university students (38, 86). Implementation intentions, a complementary strategy developed by Gollwitzer (78), extend goal setting by specifying the situational cues that will trigger goal-directed behavior (in the format “when situation X arises, I will perform behavior Y”), thereby creating a mental link between environmental cues and planned responses that can facilitate behavior initiation even when motivation is low or competing demands are high.

Situation creation and goals are partially mediated by increases in exercise self efficacy and self-regulatory capacity (38, 75). The concept of goal hierarchies, which refers to the organization of goals at different levels of abstraction from broad life values (such as health and well-being) to specific behavioral targets (such as running three times per week), is also relevant to exercise-based weight management, as research suggests that students who can clearly articulate the connections between their specific exercise goals and their broader life values are more likely to sustain their exercise behavior over time, because this hierarchical alignment provides a persistent source of meaning and motivation that can buffer against the inevitable fluctuations in day-to-day affect and energy that characterize university life (9, 18). A case study at a Brazilian university illustrated this principle by documenting the experience of a fourth-year architecture student who had repeatedly started and abandoned exercise programs throughout her university career; when a campus counselor helped her explicitly connect her exercise goals to her broader aspirations for creative energy, professional performance, and personal vitality, rather than focusing solely on weight loss numbers, she reported a fundamental shift in her relationship with exercise that resulted in sustained participation for the remaining 18 months of her degree program (66, 87). Process goals, which focus on the performance of specific behaviors rather than the attainment of specific outcomes, have also been shown to be particularly effective for university students in the early stages of an exercise program, because they provide a sense of accomplishment and competence that is within the individual’s control, unlike outcome goals such as weight loss that are influenced by many factors beyond exercise behavior alone (86, 88).

### 5.2 Motivational interviewing techniques

Motivational interviewing (MI), originally developed by Miller and Rollnick (89) as a clinical approach to facilitate behavior change in individuals experiencing ambivalence, has been increasingly adapted and applied to exercise promotion and weight management in university settings, with growing evidence supporting its effectiveness in enhancing exercise motivation, reducing ambivalence about behavior change, and improving weight-related outcomes among students who are contemplating but not yet consistently performing regular physical activity (8, 89). MI is grounded in four key principles, expressing empathy, developing discrepancy, rolling with resistance, and supporting self-efficacy, that collectively create a conversational climate in which individuals feel understood, respected, and empowered to explore and resolve their own ambivalence about change, rather than feeling pressured, judged, or lectured by a health professional or counselor (18, 89). The alignment between MI and Self-Determination Theory is particularly noteworthy, as both frameworks emphasize the importance of autonomy, competence, and relatedness in fostering lasting behavior change, and research suggests that MI may exert its positive effects on exercise behavior precisely because it satisfies these basic psychological needs by providing a non-judgmental, empathic, and autonomy-supportive relational context in which individuals can develop their own reasons for change (8, 59).

In university settings, MI has been delivered in a variety of formats including individual counseling sessions, group workshops, peer-led interventions, and even technology-mediated interactions, and research across these formats has generally shown positive effects on exercise motivation, physical activity levels, and weight management, though effect sizes vary depending on the intensity and duration of the intervention, the training and skill of the MI practitioner, and the baseline motivational profile of the student participants (8, 19). A particularly well-documented application of MI in a university weight management context occurred at a university in the southern United States, where overweight and obese students were randomly assigned to receive either three sessions of MI-based exercise counseling or three sessions of standard health education; at the end of the 10-week study period, the MI group showed significantly greater increases in weekly moderate-to-vigorous physical activity, significantly higher levels of autonomous motivation for exercise, and significantly greater reductions in body mass index compared to the health education group, with the MI group also reporting feeling more confident and less ambivalent about their ability to maintain regular exercise in the future (8, 80). These findings are consistent with a broader body of research suggesting that the quality of the interpersonal interaction between a student and a health professional or counselor may be as important as the specific content of the intervention, because the relational context in which information and advice are delivered profoundly influences how that information is received, processed, and acted upon (65, 89). The use of MI techniques has also been successfully integrated into group exercise settings, where instructors trained in MI principles create motivational climates that support autonomy, acknowledge ambivalence, and elicit students’ own reasons for exercising, resulting in higher retention rates, greater exercise enjoyment, and more positive attitudes toward physical activity compared to instructor-led programs that use more directive or prescriptive communication styles (63, 88).

### 5.3 Mindfulness and mind–body integration

Mindfulness, broadly defined as the practice of paying attention to the present moment with openness, curiosity, and non-judgment, has emerged as a promising psychological approach for enhancing exercise-based weight management among university students, with a growing body of research suggesting that mindfulness skills can improve exercise adherence by enhancing body awareness, reducing emotional reactivity, decreasing exercise-related rumination, and promoting a more accepting and compassionate relationship with one’s body and exercise experiences (90, 91). The integration of mindfulness into exercise-based weight management programs represents a significant departure from traditional approaches that emphasize willpower, self-discipline, and the suppression of unwanted thoughts and feelings, and instead proposes that the cultivation of present-moment awareness and non-judgmental acceptance can paradoxically increase behavioral regulation by reducing the internal conflict and cognitive rigidity that often undermine self-control efforts (48, 91). Research with university students has found that mindfulness is positively associated with intrinsic exercise motivation, exercise enjoyment, body appreciation, and intuitive eating, and negatively associated with exercise avoidance, emotional eating, and body dissatisfaction, suggesting that mindfulness may function as a psychological resource that supports multiple dimensions of healthy weight management simultaneously (13, 90).

Because exercise and dietary behavior are closely intertwined, mindfulness-based approaches are particularly valuable when they address eating as well as activity. Several of the case scenarios described in this review involved concurrent changes in eating behavior alongside exercise, and this synergy should be made explicit rather than left implicit. Mindful-eating and related approaches can increase awareness of internal hunger and satiety cues and reduce emotional, external, and binge eating, which complements the affect-regulation and self-compassion benefits of mindful movement (92). For university students, integrating brief mindful-eating components into exercise-based programs may therefore strengthen weight-management outcomes beyond what either exercise or dietary change achieves in isolation, although the evidence for effects on body weight specifically remains mixed and warrants cautious interpretation (92).

Mindful exercise, which involves intentionally bringing awareness to the physical sensations, movements, and breathing patterns experienced during physical activity, has been shown to enhance the positive affective response to exercise and to reduce perceived exertion during moderate-intensity exercise, both of which are associated with greater exercise adherence in university student populations (44, 48). A randomized controlled trial at a Midwestern American university compared the effects of a six-week mindful walking program with a standard walking program of identical duration and intensity among 96 sedentary female students; participants in the mindful walking group were taught to focus their attention on the sensory experience of walking, including the feeling of their feet contacting the ground, the rhythm of their breathing, and the visual and auditory details of their environment, while participants in the standard walking group walked at the same pace and duration but without mindfulness instructions; at the end of the 6-week period, the mindful walking group reported significantly greater improvements in mood, body satisfaction, and exercise enjoyment, and showed significantly higher rates of continued walking at a four-week follow-up, suggesting that the addition of mindfulness to exercise enhanced both the immediate psychological experience and the long-term behavioral sustainability of the exercise behavior (45, 91). The practice of self-compassion, which is closely related to mindfulness and involves treating oneself with kindness and understanding in the face of difficulty or perceived failure, has also been identified as an important psychological resource for university students engaged in exercise-based weight management, because self-compassion buffers against the self-critical rumination and shame that often follow perceived exercise lapses or slow weight loss progress, thereby reducing the risk of complete behavioral abandonment in response to minor setbacks (12, 13). A longitudinal study tracking self-compassion levels and exercise behavior among first-year university students found that increases in self-compassion over the first semester were associated with increases in physical activity and improvements in body image, and that students with higher self-compassion levels at the start of the year showed greater exercise consistency across the entire academic year, including during examination periods when self-regulatory resources were most depleted, highlighting the protective function of self-compassion against the psychological threats that commonly disrupt exercise behavior during the university years (13).

### 5.4 Behavioral activation and habit formation

Behavioral activation and habit formation represent complementary psychological strategies that address two distinct but equally important phases of exercise-based weight management: the initiation of exercise behavior in individuals who are currently sedentary or insufficiently active, and the automatization of exercise behavior so that it becomes a stable and self-sustaining part of the individual’s daily routine that no longer requires high levels of conscious motivation and deliberate self-regulation (42, 82). Behavioral activation, originally developed as a treatment for depression, involves the systematic scheduling and performance of valued activities that provide a sense of accomplishment and pleasure, and its application to exercise promotion is based on the premise that many students fail to exercise not because they lack motivation in a global sense but because they have not established specific behavioral routines that reliably prompt exercise at consistent times and in consistent contexts (48, 54). The behavioral activation approach to exercise emphasizes action before motivation, recognizing that waiting to “feel like” exercising is an unreliable strategy because mood and motivation are inherently fluctuating states, and that the experience of positive affect and enhanced energy that typically follows exercise can itself generate the motivation to continue exercising, thereby reversing the conventional assumption that motivation must precede action.

**Figure 4:**
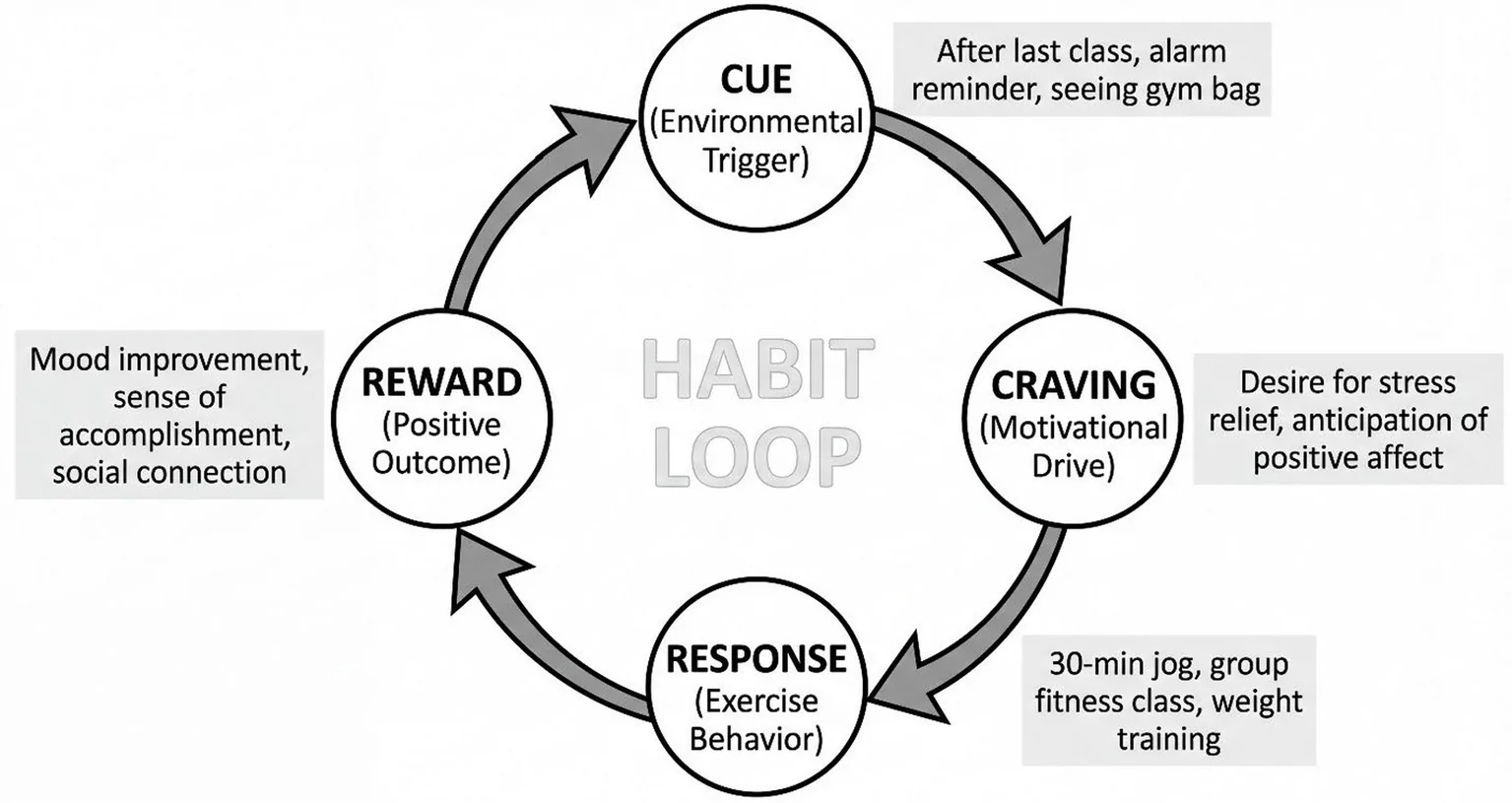
The habit formation loop for exercise behavior in university students. This figure illustrates the four-stage cyclical process of cue detection, craving activation, behavioral response, and reward experience that underlies the formation of automatic exercise habits, with examples relevant to the university student context.

Habit formation, which has been the subject of intensive research in health psychology over the past decade, refers to the process by which repeated behavioral responses in consistent contexts become automatic, cue-triggered, and relatively independent of conscious intention, and research by Lally et al. (82) has shown that the formation of exercise habits in adults typically requires between 18 and 254 days of consistent repetition, with a median of approximately 66 days, although there is substantial individual variation depending on the complexity of the behavior and the strength and consistency of contextual cues. For university students, the formation of exercise habits is both particularly challenging and particularly important: challenging because the university environment is characterized by frequent schedule changes, variable daily routines, and many competing behavioral demands; and important because robust exercise habits can protect exercise behavior against the motivational fluctuations, stress responses, and self-regulatory depletions that are endemic to academic life (42, 77). A case study from a university in New Zealand demonstrated the effectiveness of a habit-formation intervention for exercise-based weight management among a group of 12 obese first-year students who were coached to identify specific daily cues for exercise (such as returning to their dormitory after their last class), to repeat the same exercise behavior in response to these cues every day for a minimum of 8 weeks, and to reward themselves with a small non-food treat after each exercise session; by the end of the intervention, 10 of the 12 participants reported that their exercise behavior had become significantly more automatic, with reduced need for conscious decision-making and less susceptibility to mood-related fluctuations, and these participants showed significant improvements in both physical fitness indicators and body composition measurements at a six-month follow-up (42, 82). The interaction between behavioral activation and habit formation is particularly important because behavioral activation provides the initial behavioral momentum needed to establish the repetitions that habit formation requires, while habit formation provides the automaticity that sustains behavior after the deliberate effort of behavioral activation is withdrawn, creating a developmental sequence from conscious initiation through deliberate repetition to automatic execution that parallels the broader trajectory of skill acquisition and learning (77, 93) (see Figure 4).

## 6 The role of social and environmental factors

The social and environmental contexts in which university students live, study, and exercise exert profound and often underestimated influences on their exercise behavior and weight management outcomes, and understanding these contextual influences is essential for designing interventions that are not only psychologically informed at the individual level but also ecologically valid and implementable within the specific institutional and cultural settings in which students are embedded (27, 74). This section examines two key dimensions of the social-environmental context: the role of peer influence and social support networks in shaping exercise behavior, and the impact of campus environments and facility access on exercise participation and weight management among university students.

### 6.1 Peer influence and social support networks

Peer influence and social support networks are powerful determinants of exercise behavior among university students, operating through multiple mechanisms including social modeling (observing and imitating others’ behavior), social norming (perceiving what is typical or expected within one’s social group), social facilitation (performing better or more consistently in the presence of others), and emotional and instrumental support (receiving encouragement, companionship, information, and practical assistance from peers) (69, 94, 95). Research consistently shows that university students who have friends, roommates, or romantic partners who exercise regularly are significantly more likely to exercise themselves, and that this social influence operates through both motivational pathways (increasing the desire to exercise) and volitional pathways (providing practical support and accountability that facilitates the translation of motivation into action), suggesting that social relationships may be among the most potent and underutilized resources for promoting exercise-based weight management in student populations (69, 96).

The concept of exercise social identity, which refers to the degree to which an individual sees being physically active as a central and defining part of who they are as a person, is strongly influenced by peer relationships and has been identified as a significant predictor of exercise maintenance in university students, with research indicating that students who develop a strong exercise identity are more likely to sustain their exercise behavior across the challenging transitions and disruptions that characterize university life (34, 97). A case study from a South African university documented the powerful role of peer influence in a dormitory-based exercise intervention where resident advisors were trained to serve as exercise role models and to organize regular group physical activity sessions; over the course of one academic year, residents in the intervention dormitories showed significantly higher levels of exercise participation, greater weight management success, and stronger exercise social identities compared to residents in control dormitories, and qualitative interviews revealed that the social bonds formed through shared exercise experiences were perceived as one of the most valued aspects of the program, often ranked above the physical health benefits by participants (29, 95). The influence of social media on exercise behavior among university students deserves specific attention in this context, as platforms such as Instagram, TikTok, and fitness-specific applications create both opportunities for virtual social support and exercise motivation and risks of harmful social comparison, unrealistic body standards, and the promotion of extreme or unhealthy exercise and dietary practices (20, 39). Research suggests that the effects of social media on exercise behavior are highly dependent on the nature and content of social media use, with exposure to realistic, inclusive, and process-focused fitness content being associated with positive effects on exercise motivation and self-efficacy, while exposure to highly curated, appearance-focused, and competitive fitness content being associated with increased body dissatisfaction, exercise guilt, and maladaptive exercise patterns (39, 53).

### 6.2 Campus environment and access to facilities

The physical environment of the university campus, including the availability, accessibility, quality, and design of exercise facilities, recreational spaces, walking and cycling infrastructure, and the broader built environment, plays a critical role in shaping exercise behavior and weight management outcomes among university students, and this role is often overlooked in interventions that focus exclusively on individual-level psychological factors without attending to the structural and environmental contexts that facilitate or constrain individual behavior (73, 74). Ecological models of health behavior, which emphasize the multiple levels of influence on behavior ranging from intrapersonal factors to interpersonal relationships, organizational settings, community structures, and public policy, provide a useful framework for understanding how campus environmental factors interact with psychological factors to produce observed patterns of exercise behavior in university populations (74, 98). Research consistently demonstrates that proximity to exercise facilities is positively associated with exercise frequency among university students, with students whose dormitories or classrooms are located within walking distance of a gym, recreation center, or exercise-friendly outdoor space reporting significantly higher levels of physical activity than students who must travel longer distances or navigate inconvenient transportation to access exercise opportunities (29, 73).

The design and atmosphere of exercise facilities also matter for student exercise behavior, with research indicating that facilities that are perceived as welcoming, inclusive, well-maintained, and non-intimidating are associated with higher rates of student exercise participation than facilities that are perceived as crowded, dirty, dominated by experienced exercisers, or socially evaluative in atmosphere (14, 70). A comprehensive case study from a large public university in the western United States documented the effects of a campus recreation center renovation that was designed according to principles derived from environmental psychology and inclusive design; the renovated facility featured separate exercise zones for beginners and experienced exercisers, natural lighting and green plant installations, music and visual displays that created a positive and energizing atmosphere, and gender-neutral and body-inclusive messaging throughout the space; following the renovation, student exercise participation increased by 23%, with the largest increases observed among first-year students, female students, and students with higher body mass indices, precisely the populations that had been most underrepresented in the pre-renovation facility, suggesting that environmental design interventions can effectively complement psychological interventions by reducing the environmental barriers that disproportionately affect the students who are most in need of exercise support (73, 74). The broader campus environment, including the walkability of the campus, the availability of active transportation options, the presence of outdoor recreational spaces, and the integration of physical activity into the academic schedule through movement breaks or active learning approaches, also contributes to overall physical activity levels and weight management outcomes, with campuses that are designed to promote incidental physical activity (the activity that occurs as part of daily routines rather than through deliberate exercise) showing higher overall activity levels among their student populations compared to campuses that are designed primarily for automotive transportation and sedentary learning (74, 99).

## 7 Technology-mediated psychological interventions

The rapid proliferation of digital health technologies, including smartphone applications, wearable activity trackers, social fitness platforms, and gamified exercise programs, has created new opportunities for delivering psychologically informed exercise and weight management interventions to university students at scale, with the potential to overcome many of the barriers to traditional face-to-face program delivery, including limited availability of trained counselors, scheduling conflicts, cost constraints, and the stigma that some students associate with seeking help for weight-related concerns (20, 100). This section examines the evidence for technology-mediated psychological interventions in two main categories: mobile health applications and wearable devices, and gamification and digital engagement strategies (see Table 4).

**Table 4:** Technology-mediated interventions for exercise promotion in university settings.

Technology type	Psychological mechanisms	Key features	Reported outcomes	Limitations
mHealth apps (SDT-informed)	Autonomy support via choice; competence feedback; social relatedness	Personalized goals; non-comparative progress tracking; virtual communities	Significant MVPA increases over 12 weeks	Engagement declines over time; novelty effects
Wearable activity trackers	Self-monitoring; goal calibration; pattern recognition	Real-time step counts; heart rate; caloric expenditure; sleep tracking	Increased awareness of sedentary behavior; modest PA increases	Risk of compulsive self-monitoring; data overload
Gamified programs	Need satisfaction via challenge and social competition; immediate feedback	Points, badges, leaderboards, narrative levels, virtual rewards	Significant increases in daily steps and MVPA; 1,200 + participants engaged	Leaderboards can demotivate; gains may attenuate post-program
Mindfulness apps	Stress reduction; emotional regulation; body awareness enhancement	Guided meditations; stress tracking; integration with exercise routines	Reduced stress eating; improved emotional regulation; 6 kg weight loss with counselor support	Requires sustained engagement; limited personalization

### 7.1 Mobile health applications and wearable devices

Mobile health (mHealth) applications and wearable fitness devices have become ubiquitous among university students, with surveys indicating that a majority of students own at least one wearable device or have used at least one fitness application, and that these technologies are perceived as convenient, motivating, and useful tools for supporting exercise behavior and weight management (39, 101). From a psychological perspective, mHealth technologies can support exercise-based weight management through several mechanisms including self-monitoring (providing real-time feedback on activity levels, caloric expenditure, heart rate, and other exercise parameters), goal setting and progress tracking (allowing users to set specific targets and visualize their progress over time), social connection (enabling users to share their exercise data, compete with friends, and participate in virtual exercise communities), and just-in-time adaptive interventions (delivering personalized prompts, encouragement, or behavioral suggestions at moments when the user is most likely to benefit from them) (20, 100). Research has generally supported the effectiveness of mHealth interventions for increasing physical activity and promoting weight management among university students, although the evidence also indicates that the effects of these technologies are often modest in magnitude, short-lived in duration, and heavily dependent on the user’s level of engagement with the technology, with many students initially enthusiastic about fitness apps and wearables but gradually reducing their use over time as novelty effects wane and habituation sets in (39, 101).

A large-scale randomized controlled trial conducted across four university campuses investigated the effects of a smartphone-based exercise application that incorporated principles from Self-Determination Theory, including providing users with choices about exercise type and timing (autonomy support), offering personalized positive feedback based on individual progress rather than normative comparisons (competence support), and facilitating social connections with other users who shared similar exercise goals and interests (relatedness support); compared to a standard step-counting application, the SDT-informed application produced significantly greater increases in moderate-to-vigorous physical activity over the twelve-week study period, and users of the SDT-informed application also reported higher levels of intrinsic motivation, exercise enjoyment, and basic psychological need satisfaction, providing experimental evidence that the psychological design of health technology, and not merely its functional features, is a critical determinant of its effectiveness (20, 59). A case study from an East Asian university documented the experience of an international exchange student who used a combination of a wearable activity tracker and a mindfulness meditation application to manage the stress-related weight gain she was experiencing during her year abroad; the wearable device provided objective feedback that helped her calibrate her activity goals and recognize patterns in her sedentary behavior, while the mindfulness application provided guided meditations that reduced her stress eating and improved her emotional regulation capacity, and the combination of these two technologies, used in conjunction with monthly check-ins with a campus wellness counselor, supported a gradual and sustainable weight reduction of six kilograms over the academic year along with significant improvements in self-reported well-being and psychological adjustment (91, 101). However, concerns have also been raised about the potential negative effects of fitness technology, including increased body surveillance, compulsive self-monitoring, technology dependence, data privacy issues, and the exacerbation of disordered eating and exercise behaviors in vulnerable individuals, highlighting the need for thoughtful and psychologically informed design and implementation of mHealth interventions in university populations (52, 100).

### 7.2 Gamification and digital engagement strategies

Gamification, defined as the application of game design elements such as points, badges, leaderboards, challenges, narratives, and rewards to non-game contexts, has been increasingly applied to exercise promotion and weight management programs for university students, with the theoretical rationale that gamified exercise experiences can enhance intrinsic motivation, increase engagement, promote social connection, and make the exercise experience more enjoyable and psychologically rewarding than traditional exercise programming (102, 103). The psychological appeal of gamification lies in its ability to satisfy basic psychological needs as conceptualized by Self-Determination Theory: game-like choice and challenge support the need for autonomy and competence, while social competition and cooperation support the need for relatedness, and the immediate feedback provided by gamified systems satisfies the human desire for competence information and progress monitoring (18, 103). Research on gamified exercise interventions in university settings has shown promising results, with studies reporting that gamification can increase step counts, exercise session frequency, exercise duration, and overall physical activity levels among university students, although the durability of these effects beyond the gamified intervention period remains a subject of debate, with some research suggesting that gains in physical activity may attenuate when game elements are removed, raising questions about whether gamification promotes genuine internalization of exercise motivation or merely provides temporary external incentives that mask rather than resolve underlying motivational deficits (9, 103).

**Figure 5:**
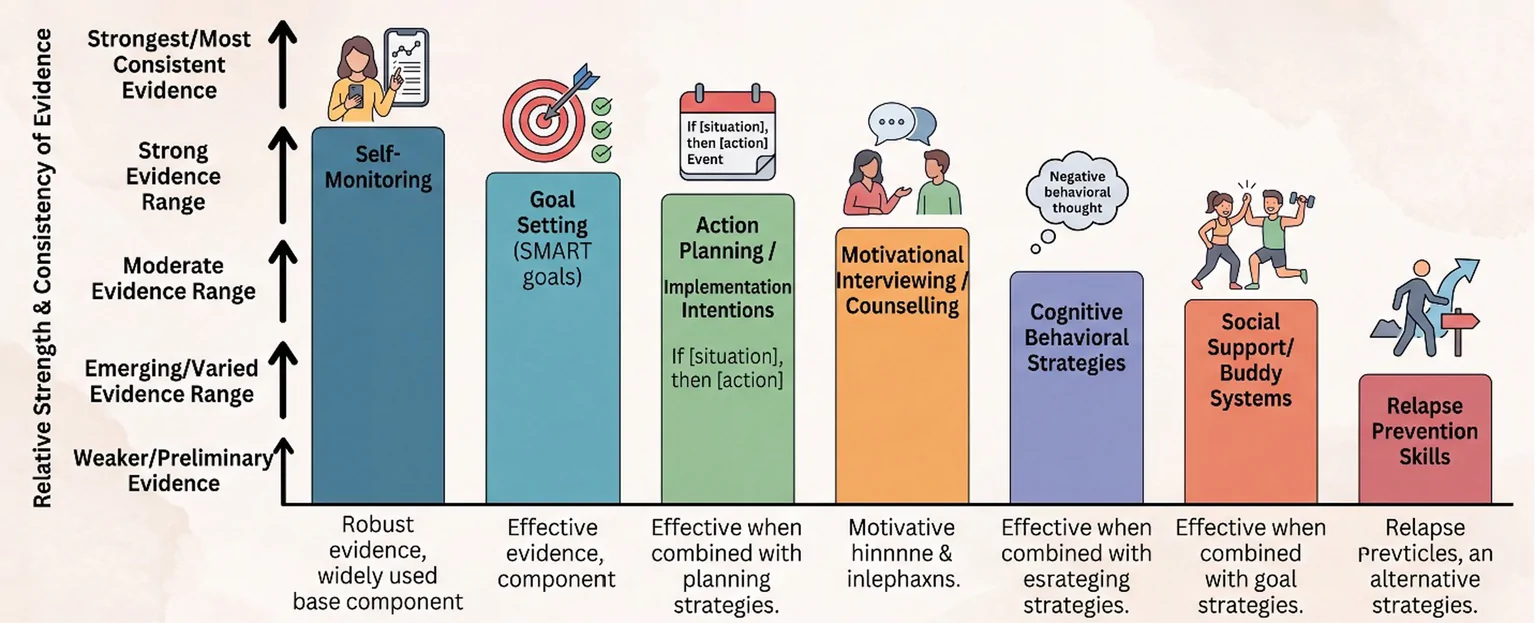
Comparative effectiveness of psychological strategies for exercise-based weight management in university students (schematic qualitative comparison). Because this is a structured narrative rather than a meta-analytic review, the figure does not report pooled or formally extracted effect sizes. Instead, it provides a schematic, qualitative ranking of the relative strength and consistency of the evidence for each strategy across the reviewed studies. The *y*-axis labels (e.g., small/medium effect benchmarks) are retained solely as a familiar visual scaffold to indicate relative ordering; they do not represent pooled Cohen’s *d* values or any other formally extracted statistic. The relative positions are illustrative only and should not be interpreted as statistically synthesized effect estimates.

A comprehensive case study from a Chinese university evaluated a semester-long gamified exercise program in which students earned points for completing daily exercise challenges, progressed through themed narrative levels, competed with peers on leaderboards, and could unlock virtual rewards and real-world privileges such as priority access to campus recreational facilities; the program attracted participation from over 1,200 students and was associated with significant increases in average daily step counts and moderate-to-vigorous physical activity compared to baseline levels, with particularly strong effects among students who had been classified as insufficiently active prior to the intervention (103). However, qualitative interviews with participants also revealed important psychological nuances, including that some students experienced the competitive leaderboard element as stressful and demotivating rather than motivating, particularly when they compared unfavorably to more active peers, and that others reported feeling extrinsically controlled by the point system rather than genuinely motivated to exercise, suggesting that the design of gamified interventions must be sensitive to individual differences in competitive orientation, self-regulation style, and motivational profile (88, 103). These findings underscore the broader principle that technology-mediated interventions, including gamified programs, are most effective when they are designed with a deep understanding of the psychological processes they are intended to influence, and when they provide sufficient flexibility and personalization to accommodate the diverse motivational profiles and preferences of university student populations (20, 59). The future development of gamified exercise interventions for university students will likely benefit from advances in artificial intelligence and machine learning that enable real-time adaptation of game elements to individual user characteristics, preferences, and behavioral patterns, creating truly personalized gamified exercise experiences that can optimize motivational impact across diverse student populations (100, 103) (see Figure 5).

## 8 Gender differences in psychological responses to exercise for weight management

**Figure 6:**
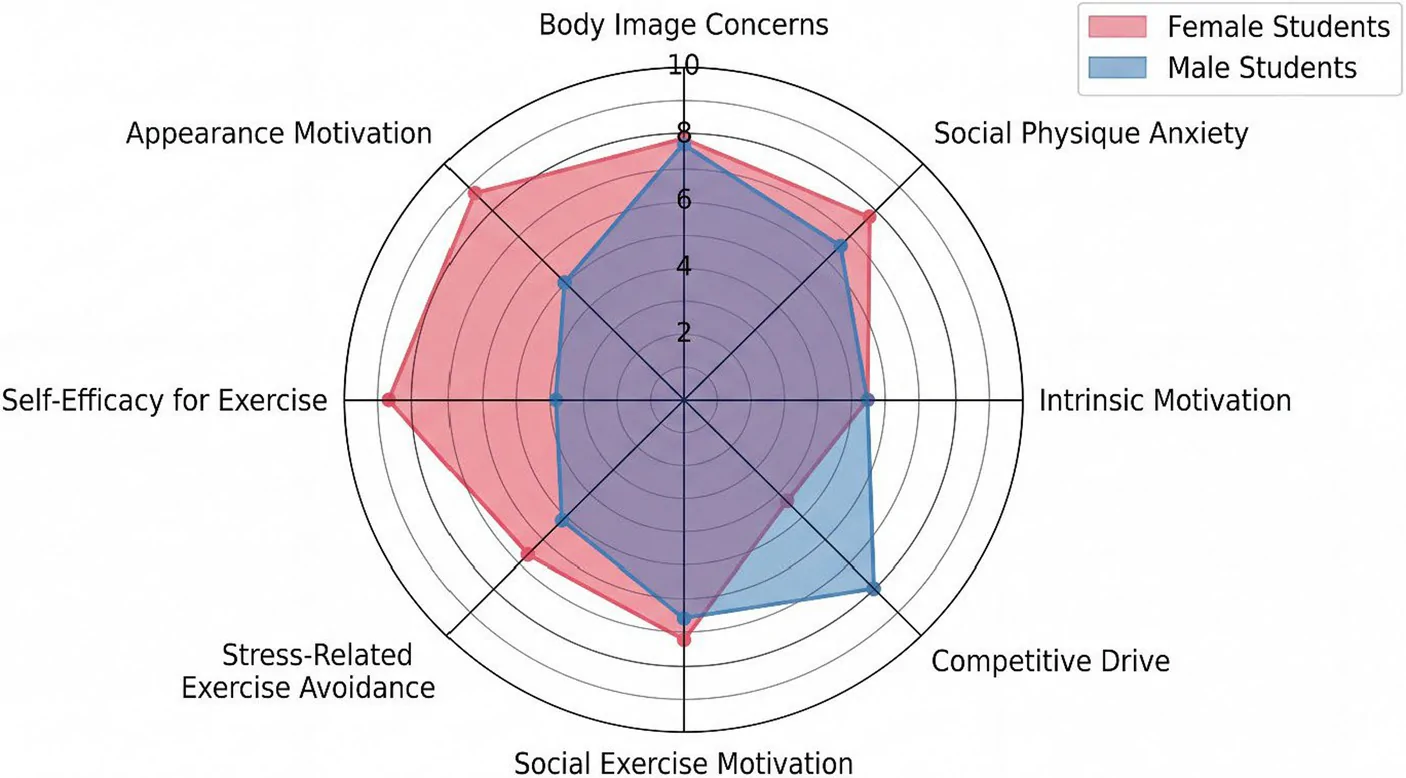
Gender differences in exercise psychology factors among university students. This radar chart compares female and male university students across eight psychological dimensions related to exercise and weight management, based on synthesized findings from reviewed studies. Scale represents relative intensity (1–10) of each factor.

Gender differences in psychological responses to exercise for weight management represent an important but often insufficiently addressed dimension of exercise promotion in university settings, as a substantial body of research indicates that male and female university students differ significantly in their motivations for exercising, their affective responses to physical activity, their vulnerability to specific psychological barriers, and their responsiveness to different intervention strategies, and failing to account for these differences in program design and delivery can reduce program effectiveness and inadvertently exclude or alienate the very students who are most in need of support (41, 83, 96). Research consistently shows that female university students are more likely than their male counterparts to report exercising primarily for weight management and appearance-related reasons, to experience social physique anxiety in exercise settings, to be influenced by social comparison and body image concerns, and to develop problematic exercise patterns such as compulsive exercise or exercise-purging behavior, while male students are more likely to report exercising for strength, fitness, and social reasons, to engage in competitive exercise and sport, and to underestimate health risks associated with extreme exercise or supplement use (28, 83, 104). These gender differences are not biologically determined but rather reflect the internalization of culturally constructed gender norms regarding body ideals, physical competence, and the social meaning of exercise, and they have important implications for the design of exercise-based weight management programs that are inclusive, equitable, and responsive to the diverse needs and experiences of all university students (41, 53) (see Figure 6).

A large cross-sectional study at Norwegian universities found that female students reported significantly higher levels of body dissatisfaction and exercise avoidance than male students at equivalent body mass index levels, and that the relationship between body dissatisfaction and exercise avoidance was substantially stronger among female students, suggesting that body image is a more powerful psychological barrier to exercise for women than for men in university settings (14, 28). A case study from a private university in the Middle East described a gender-responsive exercise program that was developed after campus health surveys revealed that female students were significantly less likely to use campus fitness facilities than male students, despite reporting similar levels of desire to exercise; the program created women-only exercise spaces, offered culturally sensitive exercise options, trained female exercise leaders to provide empathic and body-affirming feedback, and incorporated body appreciation and self-compassion workshops into the exercise programming; over two semesters, female participation in campus exercise programs increased substantially, and participating female students reported significant improvements in exercise self-efficacy, body image, and weight management self-regulation, demonstrating the value of gender-responsive program design (see Table 5).

**Table 5:** Gender differences in exercise psychology among university students.

Psychological factor	Female students	Male students
Primary exercise motivation	Weight management, appearance, stress relief, flexibility	Strength, fitness, competition, social enjoyment, muscle building
Body image concerns	Higher body dissatisfaction; thinness-oriented ideals; stronger link between body image and exercise avoidance	Lower body dissatisfaction overall; muscularity-oriented ideals; risk of muscle dysmorphia
Social physique anxiety	Significantly higher levels; stronger barrier to gym use and group exercise participation	Lower levels overall; less influence on exercise facility use
Self-efficacy for exercise	Generally lower, especially for strength training and competitive sports	Generally higher; may overestimate capacity leading to injury risk
Social media influence	Greater susceptibility to thin-ideal content; increased body comparison	Greater susceptibility to muscular-ideal content; supplement and performance enhancement interest
Help-seeking behavior	More willing to seek support for weight concerns and mental health	Cultural reluctance to seek help; weight concerns seen as inconsistent with masculine identity
Preferred exercise context	Group classes; low-competitive settings; mind–body activities; women-only spaces	Competitive sports; weight rooms; individual training; team-based activities

## 9 Long-term psychological outcomes of exercise-based weight reduction

The long-term psychological outcomes of exercise-based weight reduction among university students extend far beyond the immediate physical changes in body composition and encompass a broad range of psychological benefits including sustained behavior change, improved mental health, enhanced self-concept, greater resilience, and improved quality of life that can persist long after the university years have ended (105, 106). Understanding these long-term outcomes is important not only for motivating students to engage in exercise in the present but also for informing university policies and programs that aim to promote lifelong health and well-being through the establishment of healthy behavioral patterns during the critical developmental period of young adulthood.

### 9.1 Sustained behavior change and weight maintenance

Sustained behavior change and the maintenance of exercise-related weight reduction over the long term represent the ultimate goal of any exercise-based weight management intervention, and achieving this goal requires the successful transition from externally motivated and deliberately regulated exercise behavior to internally motivated and habitually maintained physical activity that is experienced as a valued and integral part of one’s identity and lifestyle (9, 42, 82). Research indicates that the majority of individuals who achieve initial weight loss through exercise regain some or all of the lost weight within two to 5 years, a finding that has led to increasing recognition that the psychology of weight maintenance is distinct from the psychology of weight loss and may require different intervention strategies, including a greater emphasis on habit formation, identity change, flexible self-regulation, and the cultivation of intrinsic exercise motivation (42, 93). For university students, the transition from university to post-university life represents a particularly high-risk period for exercise relapse and weight regain, because this transition typically involves major changes in daily routine, social network, residential environment, and occupational demands that can disrupt even well-established exercise habits, and because the institutional supports for exercise that may have been available during university (campus recreation facilities, organized intramural sports, peer exercise groups) are often no longer accessible after graduation (1, 6).

Longitudinal research tracking university students from enrollment through graduation and into early career has identified several psychological factors that predict the maintenance of exercise behavior and weight management across these transitions, including the strength of exercise identity (the degree to which being physically active is integrated into one’s self-concept), the level of autonomous motivation for exercise (exercising because one wants to rather than because one feels pressured to), the robustness of exercise habits (the degree to which exercise has become automatic and cue-triggered), and the breadth and flexibility of one’s exercise repertoire (the ability to adapt exercise behavior to different contexts, schedules, and physical environments) (34, 42, 97). A case study from a longitudinal cohort study at a Canadian university followed a group of students who had participated in a comprehensive exercise-based weight management program during their second year and tracked their exercise behavior, body weight, and psychological well-being over the following 5 years; the study found that the students who maintained their exercise behavior and weight loss most successfully were those who had developed strong exercise identities, had transitioned from controlled to autonomous exercise motivation during the intervention, and had established flexible exercise habits that could accommodate the changing demands and contexts of their post-university lives, while students who had exercised primarily for externally regulated reasons and had not developed habitual exercise routines were significantly more likely to relapse into sedentary behavior within the first year after graduation (1, 9, 97). These findings have important implications for the design of university exercise programs, suggesting that programs should aim not only to increase exercise behavior and reduce weight during the program period but also to foster the psychological resources, identity, autonomous motivation, habit, flexibility, that will enable students to maintain their exercise behavior across the inevitable transitions and disruptions that characterize the post-university years.

### 9.2 Mental health benefits beyond weight loss

The mental health benefits of regular exercise extend well beyond its effects on body weight and represent some of the most consistently documented and clinically significant outcomes of physical activity in university student populations, with research demonstrating that regular exercisers report lower levels of depression, anxiety, and perceived stress and higher levels of self-esteem, life satisfaction, and overall psychological well-being compared to their sedentary peers, even after controlling for baseline mental health status and other potential confounding variables (55–57). The mechanisms through which exercise produces mental health benefits are multiple and include both biological pathways (such as the release of endorphins and endocannabinoids, the normalization of hypothalamic–pituitary–adrenal axis function, the promotion of neurogenesis and neuroplasticity, and the improvement of sleep quality) and psychological pathways (such as enhanced self-efficacy, increased sense of mastery and control, improved body image, greater social connection, and the development of more adaptive coping strategies), and these mechanisms are likely to operate synergistically to produce the overall mental health benefits that are observed in physically active university students (81, 85, 107).

For university students engaged in exercise-based weight management, the mental health benefits of exercise serve a critical dual function: they improve well-being directly by reducing psychiatric symptoms and enhancing positive psychological states, and they also improve weight management outcomes indirectly by reducing the emotional eating, stress-related sedentary behavior, and motivational impairments that are associated with poor mental health and that can undermine weight loss efforts (49, 55). A comprehensive case study from an Australian university wellness program documented the integrated physical and mental health outcomes of a cohort of 45 students who participated in a 12-week combined exercise and psychological support program; participants who completed the program showed significant improvements in both physical measures (aerobic capacity, body composition, muscular strength) and psychological measures (depressive symptoms, anxiety levels, self-esteem, academic stress, and sleep quality), and these improvements were significantly correlated with each other, with greater physical improvements associated with greater psychological improvements and vice versa, supporting the theoretical proposition that physical and mental health are deeply interconnected and that interventions addressing both domains simultaneously can produce synergistic benefits that exceed the effects of either domain addressed in isolation (11, 106). The concept of flourishing, which refers to a state of optimal psychological functioning characterized by positive emotions, engagement, meaning, positive relationships, and achievement, has been increasingly applied to the study of exercise and well-being in university students, with research suggesting that regular exercise contributes to flourishing not only by reducing negative mental health symptoms but also by actively promoting positive psychological resources such as vitality, gratitude, optimism, and purpose in life that enable students to thrive rather than merely survive during their university years and beyond (50, 56, 105).

## 10 Recommendations and future directions

Based on the comprehensive review of psychological insights presented in this article, several evidence-based recommendations can be formulated for university stakeholders, health professionals, counselors, and researchers seeking to promote effective exercise-based weight reduction among university students, and these recommendations span multiple levels of influence from individual psychological interventions to institutional and environmental changes that create supportive contexts for student health behavior (19, 74). At the individual level, interventions should prioritize the development of intrinsic motivation for exercise by creating opportunities for autonomy, competence, and relatedness that align with Self-Determination Theory principles, rather than relying on external incentives, appearance-based appeals, or fear-based messaging that may produce short-term compliance but are unlikely to sustain long-term behavior change (9, 59). Goal-setting interventions should emphasize specific, process-focused, and hierarchically organized goals that connect daily exercise behaviors to broader personal values and life aspirations, and should incorporate implementation intentions that specify the precise situational cues and behavioral responses that will guide exercise behavior in the context of competing daily demands (38, 78, 86). Motivational interviewing techniques should be integrated into campus exercise counseling and health promotion services as a means of addressing ambivalence, building autonomous motivation, and providing the empathic and supportive relational context that facilitates lasting behavior change (8, 89). Mindfulness and self-compassion training should be offered as adjunctive components of exercise-based weight management programs, as these skills can enhance exercise enjoyment, reduce body image distress, improve emotional regulation, and protect against the self-critical rumination that often follows perceived exercise setbacks or slow weight loss progress (13, 91). Habit formation strategies should be emphasized from the earliest stages of exercise program participation, with a focus on environmental cue identification, behavioral consistency, and the gradual reduction of conscious effort required to maintain exercise behavior over time (42, 82).

At the institutional level, universities should invest in the design of exercise environments that are welcoming, inclusive, and psychologically safe for students of all body types, fitness levels, and exercise experiences, recognizing that the physical and social atmosphere of exercise facilities can be as influential as individual psychological interventions in determining whether students adopt and sustain exercise behavior (73, 74). Gender-responsive programming that addresses the specific psychological barriers and motivational profiles of female, male, and gender-diverse students should be developed and evaluated, with attention to the role of body image, social physique anxiety, cultural norms, and identity-related factors in shaping exercise behavior across different student subgroups (28, 104). Technology-mediated interventions, including SDT-informed mobile applications, wearable activity trackers, and thoughtfully designed gamified exercise programs, should be integrated into campus wellness infrastructure as scalable and cost-effective tools for reaching large numbers of students with personalized psychological support for exercise and weight management (20, 103). Research in this domain should continue to move toward more rigorous longitudinal designs that can capture the dynamic and reciprocal relationships between psychological factors, exercise behavior, and weight outcomes over the extended time periods that are relevant to behavior change maintenance and long-term health, and toward more inclusive and diverse sampling strategies that can address the significant gaps in our understanding of exercise psychology among underrepresented student populations including international students, students with disabilities, students from low socioeconomic backgrounds, and first-generation college students (19, 26). Future research should also examine the optimal timing, duration, intensity, and delivery format of psychological interventions for exercise-based weight management, with attention to the question of whether certain intervention components are more effective at particular stages of the behavior change process and whether booster sessions or ongoing support are necessary to sustain intervention effects beyond the initial program period (59, 93).

Alongside effectiveness, the cost-effectiveness of psychologically informed exercise strategies warrants greater attention than it has typically received in this literature. Given the scale of physical inactivity and its downstream economic burden (98), universities must weigh the resource intensity of different approaches, for example, individually delivered motivational interviewing or counselor-guided goal setting versus lower-cost, scalable digital tools such as need-supportive mobile applications and thoughtfully designed gamified programs. Economic evaluations of behavioral physical-activity interventions suggest that comparatively brief, low-intensity approaches can be cost-effective relative to usual care, although longer-term cost and benefit data remain limited (108). A fuller consideration of cost-effectiveness, including implementation costs, reach, and the maintenance of effects over time, should therefore be a routine component of program planning and of future research in this area.

Finally, the evidence synthesized here varies considerably in strength and consistency across study designs. Much of the literature relies on cross-sectional and short-term designs, samples are frequently drawn from convenience populations, and findings for several constructs (for instance, the specific consequences of introjected regulation, or the effects of some technology-mediated tools) are mixed rather than uniformly positive (7, 9). These limitations, together with the illustrative nature of the case vignettes and the non-systematic scope of this review, indicate that the conclusions should be read as an integrative interpretation of a heterogeneous evidence base rather than as definitive quantitative estimates.

## 11 Conclusion

This comprehensive review has examined the rich and complex landscape of psychological insights that are relevant to understanding and promoting effective weight reduction through exercise among university students, a population that faces unique challenges and opportunities for health behavior change during a critical developmental period. The evidence reviewed in this article consistently supports the central thesis that the psychological dimensions of exercise, including motivation, self-efficacy, emotional regulation, cognitive appraisal, social influence, body image, habit formation, and identity, are at least as important as the physiological dimensions in determining whether university students successfully adopt, maintain, and benefit from exercise-based weight management strategies (9, 17, 59). Theoretical frameworks including Self-Determination Theory, Social Cognitive Theory, and the Theory of Planned Behavior provide complementary and empirically supported lenses through which these psychological processes can be understood and targeted in intervention design, and the integration of insights from these frameworks with emerging evidence on mindfulness, habit formation, gamification, and technology-mediated interventions offers promising avenues for developing more effective, engaging, and sustainable exercise programs for university students (18, 42, 103).

The case studies integrated throughout this review illustrate the practical relevance of these theoretical and empirical findings, demonstrating how individual students’ exercise and weight management trajectories are shaped by psychological factors that interact with social, environmental, and institutional contexts in complex and often unpredictable ways (8, 74). These case studies also highlight the importance of personalized and holistic approaches to exercise-based weight management that recognize the uniqueness of each student’s psychological profile, motivational landscape, and contextual circumstances, rather than applying standardized exercise prescriptions that may be effective for some students but ineffective or even counterproductive for others (64, 89). As universities increasingly recognize their responsibility to support the holistic health and well-being of their students, the psychological insights reviewed in this article can serve as a valuable foundation for the design and implementation of exercise-based weight management programs that are scientifically grounded, psychologically informed, culturally responsive, and genuinely empowering for the diverse student populations they aim to serve (11, 19). The ultimate vision articulated by this review is one in which the principle of “mind over matter” is not interpreted as a call for willpower or self-denial, but rather as a recognition that the cultivation of psychological well-being, intrinsic motivation, self-compassion, and meaningful social connection creates the mental foundation upon which lasting physical health behaviors, including exercise and weight management, can be built and sustained throughout the university years and beyond (62, 105, 106).
